# Biological Dynamics Markup Language (BDML): an open format for representing quantitative biological dynamics data

**DOI:** 10.1093/bioinformatics/btu767

**Published:** 2014-11-19

**Authors:** Koji Kyoda, Yukako Tohsato, Kenneth H. L. Ho, Shuichi Onami

**Affiliations:** ^1^Laboratory for Developmental Dynamics, RIKEN Quantitative Biology Center, Kobe 650-0047, Japan and; ^2^National Bioscience Database Center, Japan Science and Technology Agency, Tokyo 102-0081, Japan

## Abstract

**Motivation:** Recent progress in live-cell imaging and modeling techniques has resulted in generation of a large amount of quantitative data (from experimental measurements and computer simulations) on spatiotemporal dynamics of biological objects such as molecules, cells and organisms. Although many research groups have independently dedicated their efforts to developing software tools for visualizing and analyzing these data, these tools are often not compatible with each other because of different data formats.

**Results:** We developed an open unified format, Biological Dynamics Markup Language (BDML; current version: 0.2), which provides a basic framework for representing quantitative biological dynamics data for objects ranging from molecules to cells to organisms. BDML is based on Extensible Markup Language (XML). Its advantages are machine and human readability and extensibility. BDML will improve the efficiency of development and evaluation of software tools for data visualization and analysis.

**Availability and implementation:** A specification and a schema file for BDML are freely available online at http://ssbd.qbic.riken.jp/bdml/.

**Contact:** sonami@riken.jp

**Supplementary Information:** Supplementary data are available at *Bioinformatics* online.

## 1 Introduction

With the rapid progress in live-cell imaging and modeling techniques, quantitative spatiotemporal dynamics of biological objects such as molecules, cells and organisms can be obtained from experimental measurements and computer simulations (Keller, 2013; Mogilner *et al*., 2006; Oates *et al*., 2009). Such quantitative data can provide us with new opportunities to analyze biological dynamics through various types of computational methods; these data would also provide a rich resource for understanding the mechanisms of biological systems.

A wide variety of quantitative biological dynamics data can be directly obtained from experimental measurements by using live-cell imaging and digital image processing; for example, cell division dynamics in *Caenorhabditis elegans* can be extracted from four-dimensional (4D) microscopic images (Bao *et al*., 2006; Giurumescu *et al*., 2012; Kyoda *et al*., 2013; Sarov *et al*., 2012). Similarly, quantitative data can be obtained for embryogenesis in *Drosophila melanogaster* (Keller *et al*., 2010; Supatto *et al*., 2009) and zebrafish (Keller *et al*., 2008) and for behavioral dynamics in adult *C.elegans* (Cronin *et al.*, 2005; Yemini *et al*., 2013). Quantitative data can also be obtained from computer simulations, e.g. single-molecule dynamics in *Escherichia coli* (Arjunan and Tomita, 2010) and microtubule-dependent nuclear dynamics in *C.elegans* embryos (Kimura and Onami, 2005). Although most of these data are publicly available, it is often difficult to reuse them because of their intricate structure and the lack of detailed explanations of their formats.

Although various software tools have been developed independently for different types of quantitative data, they are often not compatible with each other as they tend to use different data formats. For example, dataset-specific software tools have been developed for visualization of cell division dynamics in *C.elegans* embryos (Boyle *et al*., 2006; Hamahashi *et al*., 2005; Kyoda *et al*., 2013), but they are not interchangeable and it is difficult to reuse these software tools to visualize different datasets. Similarly, separate software tools have been developed to analyze the dynamics of *C.elegans* and zebrafish embryos (Keller *et al*., 2008; Moore *et al*., 2013).

One of the solutions is to develop a unified format for representing quantitative biological dynamics data. Similar problems with the data formats existed in the field of systems biology, and various data formats have been developed to solve the problems. CellML is designed to represent biological models using algebraic and differential equations and associated meta-information (Hedley *et al*., 2001). SBML is designed for representation and exchange of biochemical network models (Hucka *et al*., 2003). Both CellML and SBML are based on Extensible Markup Language (XML) (http://www.w3.org/TR/2008/REC-xml-20081126/). FieldML is used to represent parameterized spatial fields such as finite element method models (Britten *et al*., 2013). It is based on XML and also supports HDF5, a hierarchical binary format that allows the data to be accessed more efficiently. Combined with CellML, FieldML can provide a complete vocabulary for describing models at a range of resolutions from the cellular level to the whole-organ level.

MAGE-ML (Spellman *et al.*, 2002), MAGE-TAB (Rayner *et al.*, 2006), MINiML (Barrett *et al.*, 2007), mzML (Martens *et al.*, 2011) and BioSignalML (Brooks *et al.*, 2011) are formats for representing experimental results. MAGE-ML, MAGE-TAB and MINiML are formats for sharing microarray data; they follow the MIAME guidelines (Brazma *et al.*, 2001). MAGE-ML and MINiML are both XML-based formats. The advantage of MAGE-ML is that it allows easy development of database applications, whereas that of MINiML is its simplicity. MAGE-TAB is a simple spreadsheet-based format for representing microarray data and associated meta-information to address the needs of experimental biologists. mzML is an open format using XML and binary formats for storage and exchange of mass spectrometry data. It allows storage of both spectral and chromatographic data as binary format data encoded into base 64 strings and includes an index to allow random access to the data. BioSignalML uses the Resource Description Framework (RDF) for encoding and storing of biomedical signals such as electrocardiograms and associated meta-information. It stores the signals as a sequence of time-varying data points in their native binary formats, e.g. HDF5.

SBRML (Dada *et al.*, 2010) is an XML-based format for representing both experimental and simulation results. It focuses on associating systems biology data, such as microarray data, with cellular models. It also supports both spreadsheet-like data and multidimensional data cubes. However, none of the formats mentioned above were designed to represent three-dimensional (3D) spatial and temporal dynamics of biological objects. Representing quantitative biological dynamics data using existing formats would be difficult and inefficient. Therefore, development of a new data format is needed.

In this study, we developed an open format for representing quantitative biological dynamics data, Biological Dynamics Markup Language (BDML). BDML can describe a wide variety of spatiotemporal dynamics of biological objects at different levels, from molecules to cells to organisms. The biological objects are represented as predefined geometric entities such as points, lines, circles, spheres, faces and combinations of the above. BDML is based on XML. The BDML format is both machine and human readable, which should enable computational biologists to efficiently develop and evaluate software tools. It is also extensible, which should enable flexible future support for new types of quantitative biological dynamics data. The current version of BDML is 0.2. We expect that the BDML framework will dramatically accelerate the analysis of quantitative biological dynamics data, which in turn will allow us to gain a better understanding of the mechanisms of biological systems.

## 2 Methods

### 2.1 Overview

#### 

BDML is based on XML, which is in turn a derivative of the Standard Generalized Markup Language (SGML). SGML is an international standard for information processing. It defines a set of markup tags to describe the document structure and other attributes. XML provides a subset of SGML markup tags and is now widely accepted by the bioinformatics community as a standard data format (Achard *et al*., 2001).

A specification or grammar written in XML is called a schema. A schema defines an XML document, allowing easy validation of the syntax and making the document self-contained, i.e. an XML document does not require additional files or documents to describe the data structure within the document.

Our first requirement for a unified data format was sufficient machine and human readability to allow computational biologists to accelerate software development and evaluation. The machine readability of XML with a large number of open libraries and Application Programming Interfaces (APIs) enables efficient software development and evaluation. The human readability of XML allows experimental biologists to easily access and understand the content of quantitative data. Our second requirement was flexibility and extensibility. The format needs to be flexible enough to allow future extension to accommodate new types of quantitative data. The extensibility of XML enables the BDML format to support flexible data extension. Therefore, XML was chosen as the basis for BDML.

A BDML file usually consists of six top-level elements: info, ontology, summary, contact, methods and data. The info element provides information about the BDML file, whereas the ontology, summary, contact and methods elements represent meta-information of the quantitative data. The data element contains the quantitative data obtained from either experimental measurements or computer simulations.

##### Info

A short description of the content of the BDML file. It includes a unique identifier for each BDML file and details of its license.

##### Ontology

A description of the associations between terms in BDML and those from external ontology sources.

##### Summary

A short summary of the quantitative biological dynamics data described in the BDML file.

##### Contact

Detailed information about the corresponding author of the BDML file. Contact name, affiliation and e-mail address must be included with each BDML file.

##### Methods

A description of the method used to obtain the quantitative data. The description should provide enough detail to allow another person or group to reproduce the quantitative data from the original sources, e.g. microscopic images.

##### Data

A description of the spatiotemporal quantitative data.

**Fig. 1.**
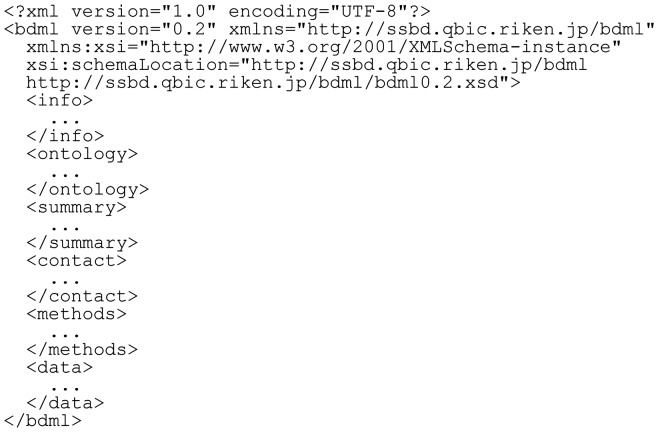
The skeleton of a BDML file, showing all top-level elements

The BDML data format begins with an XML declaration (Fig. 1). The next element, bdml, contains the top-level elements in the following order: info, ontology, summary, contact, methods and data. Most elements in BDML are derived from a single abstract base type, BDBase, which supports attaching metadata, notes and annotations to the elements. The series and set elements can be used instead of data. The series element is used when the data are too large and we want to represent a dataset in a series of data files. For example, this element can be used to divide a dataset into more than one data file, each corresponding to the data within a specific time frame. The set element is used when we want to treat more than one data file derived from related but separate experiments or simulations as a set. For example, this element can be used to describe a set of experimental measurements in a single published work. The series and set elements encapsulate a list of unique identifiers (see the description in the section 2.2.1.).

### 2.2 The elements of BDML

A BDML file can contain all the quantitative data and associated meta-information in a single file. In this section, we describe each top-level element of BDML with the help of examples. To make it easier for readers to understand the BDML format, these descriptions focus on major BDML elements and omit many details. A schema and specification for BDML are available at http://ssbd.qbic.riken.jp/bdml/.

#### 2.2.1 Info element

**Fig. 2.**
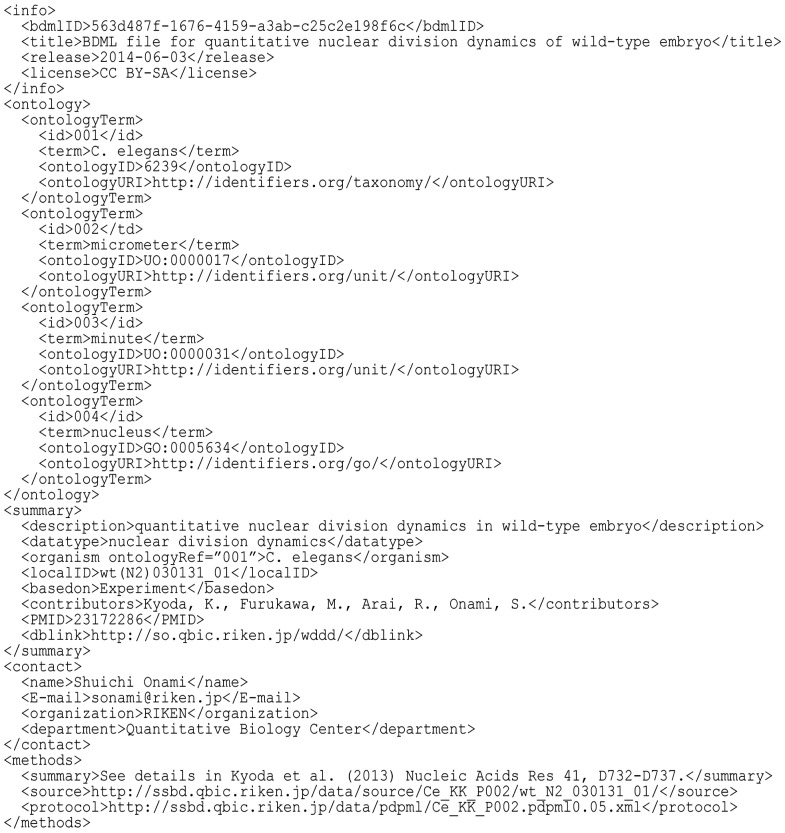
Examples of the info, ontology, summary, contact and methods elements

The info element describes the content of the BDML file. Each BDML file has a unique identifier bdmlID (Fig. 2), which is used to identify the file when it is shared or exchanged. This identifier is defined by a Universally Unique Identifier (http://tools.ietf.org/html/rfc4122), which is a standard identifier used in most software tools. It can be generated without central coordination, thus allowing the user to generate his or her own identifier without worrying that someone else will generate the same identifier. License information such as the Creative Commons licenses (http://creativecommons.org/licenses/) should be explicitly described to avoid unnecessary conflicts.

#### 2.2.2 Ontology element

**Fig. 3.**
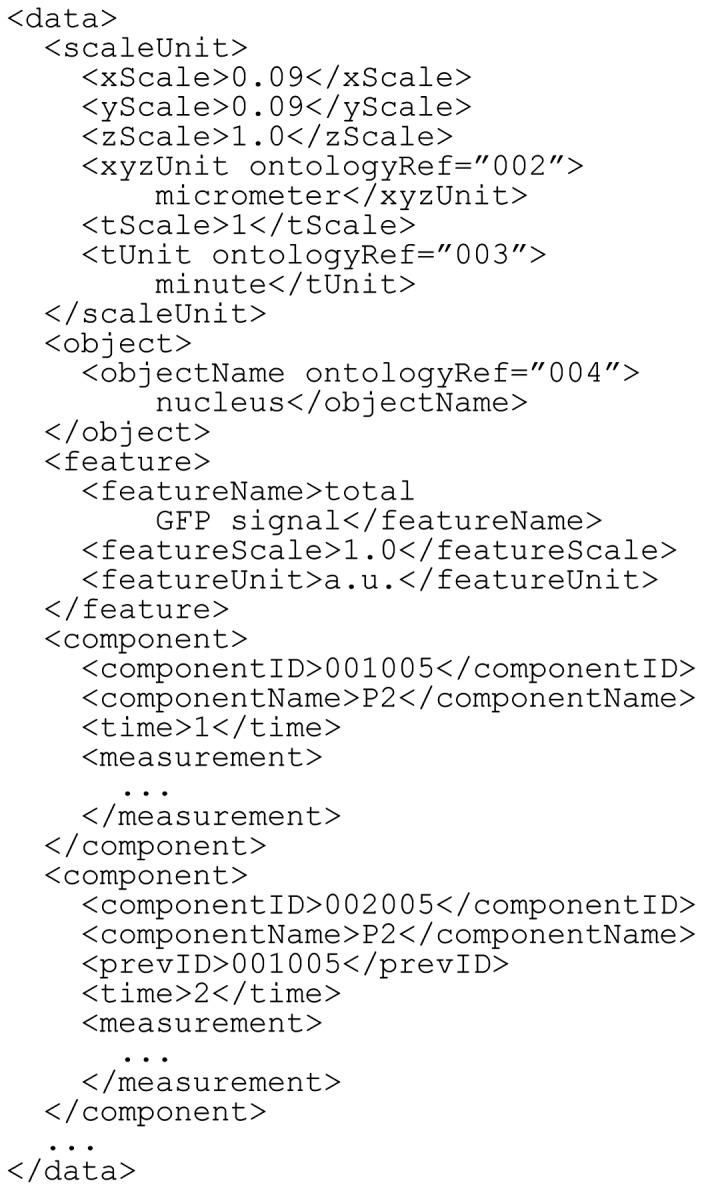
An example of the data element

The ontology element associates terms in BDML with those from external ontology sources (Fig. 2). The use of ontological references ensures unambiguous interpretation of information in a BDML file. Each term in BDML can be associated with a term from different ontology sources in the ontologyTerm element. The elements id and term represent a unique identifier for the ontology term and the term itself, respectively. The elements ontologyID and ontologyURI represent an accession identifier and a unique identifier of the ontology source, respectively. The ontologyRef attribution can be used in the following elements: datatype, organism (see section 2.2.3 and Fig. 2), objectName, xyzUnit, tUnit and featureUnit (see section 2.2.6 and Fig. 3). The ontologyRef attribution refers to id defined in the ontologyTerm element (Figs 2 and 3). The ontology element is optional in the current version of BDML (0.2). Although biologists producing quantitative biological dynamics data are in the best position to annotate their data using ontological terms, most biologists are not well versed in the use of ontologies.

#### 2.2.3 Summary element

The summary element provides a concise description of the quantitative data in the BDML file. The datatype element is used to indicate what biological process was targeted to obtain the quantitative data. A target organism should be indicated according to National Center of Biotechnology Information (NCBI) taxonomy (NCBI Resource Coordinators, 2014) (Fig. 2). The localID element can be used to link bdmlID to the internal identifier names of each author in the laboratory. The basedon element indicates whether the data are derived from an experimental measurement or computer simulation. Detailed information on a published paper or database can be included in the summary element.

#### 2.2.4 Contact element

The contact element describes detailed information about the corresponding author of the BDML file. Contact name, e-mail address and affiliation of the corresponding author should be listed (Fig. 2).

#### 2.2.5 Methods element

The methods element describes the procedure used to produce the quantitative spatiotemporal data described in the BDML file. This element is designed to enable reproduction of the quantitative data from the original sources by providing references to previous work (Waltemath *et al.*, 2011). This element includes two hyperlinks that are defined as Uniform Resource Identifiers. The first link points to the original sources such as microscopic images (for an experimental measurement) or files of a mathematical model (for a computer simulation). The second link points to a description of the procedure (which can be a web page or file) used to obtain the quantitative spatiotemporal data from the original sources (Fig. 2). As an alternative description of such procedure, we prepared an XML-based language named Procedure for Data Processing Markup Language (PDPML) (Supplementary Section S1). A schema and specification for PDPML are available at http://ssbd.qbic.riken.jp/pdpml/.

#### 2.2.6 Data element

The data element contains the quantitative spatiotemporal data (Fig. 3). It has four sub-elements: scaleUnit, object, feature and component. The scale and units of the coordinates and time are defined in scaleUnit. For experimental spatiotemporal datasets, spatial information is often recorded as a set of pixel coordinates measured directly from the microscopic images, whereas time is usually considered as a sequence of regular time frames. The scaleUnit element can be used to convert each set of pixel coordinates and time frames into the actual positions and actual time, respectively. The scale factors for the *x*, *y* and *z* dimensions and time can be defined separately. The actual positions can be directly described by setting the scale factors for the *x*, *y* and *z* dimensions to 1.0. In the same way, the actual time or discontinuous time can be directly described when the scale factor for time is set to 1.0. If a dataset has only the *x* and *y* dimensions, the scale factor for the *z* dimension should be set to zero. The units of the coordinates and time should be selected from the units predefined in the BDML schema (http://ssbd.qbic.riken.jp/bdml/).

**Fig. 4.**
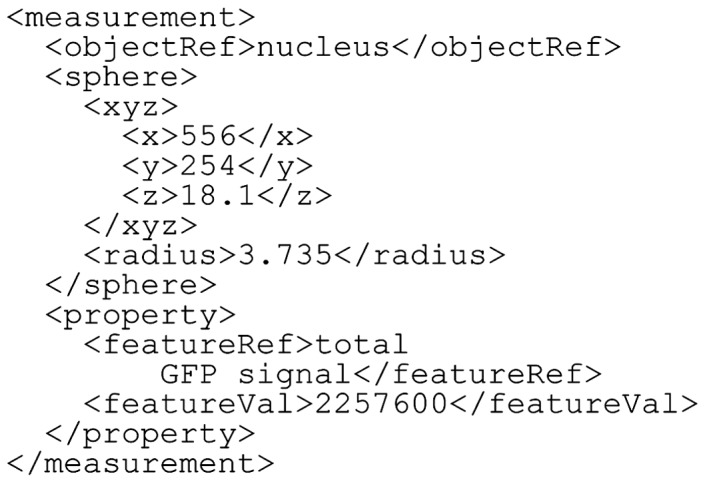
An example of the measurement element. In this example, the units are in micrometers, and the scale factors for the *x*, *y* and *z* dimensions are 0.9, 0.9 and 1.0, respectively (see Fig. 3)

The object element specifies the types of objects whose dynamics are described in the BDML file (Fig. 3); the number of object types is unlimited. The objectName element can be referred to by the objectRef element in measurement (which is a sub-element in component; see the description of measurement below; Fig. 4). The feature element specifies the types of objects’ features (Fig. 3). There can be more than one feature for an object. Each feature has its quantitative value. The scale factor and the unit should be defined in featureScale and featureUnit; featureName can be referred to by the featureRef element in measurement (see the description of measurement below; Fig. 4).

The component element describes a collection of objects and their spatial information at a given time point (Fig. 3). Each component must be separated in time (i.e. have a different time frame); therefore, it must contain the time element. The value in the time element is the elapsed time from the beginning of a microscopic recording in an experimental measurement. In a computer simulation, it is the elapsed time from the beginning of the simulation. Each component also has a unique identifier, componentID. The prevID element can be used to define a reference to or connectivity with another component at a previous time point, if applicable (Fig. 3). There can be more than one prevID, e.g. in the case of object fusion. The name of the component can also be given in componentName. Each component requires at least one measurement to describe spatial information of the objects.

**Fig. 5.**
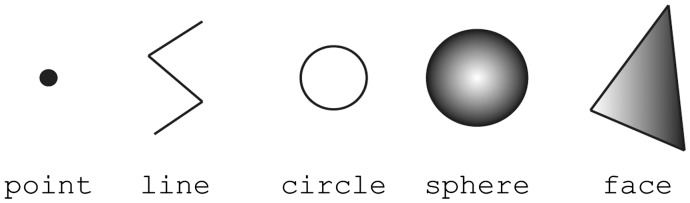
A graphical representation of spatial information of an object. A point is defined as 3D coordinates. A line is defined as a series of connected line segments. A circle or sphere is defined as a center and radius of a circle or sphere, respectively. A face is defined as a closed polygonal chain with three points

The measurement element represents spatial information of an object (Fig. 4). Each measurement corresponds to the coordinates of the object’s position. objectRef refers to an object defined in the object element. An object can be described by the following five types of entities: a set of points, a set of lines, a circle, a sphere, a set of faces or by a combination of the five types (Fig. 5). Examples of representation of spatial information are given in section 3.1. The object’s features can be described under the property element. The featureRef sub-element in the property element refers to a feature defined in the feature element. The numerical value of the feature can be recorded in featureVal.

#### 2.2.7 Unit definitions

The units for spatial and feature information are predefined under the UnitKind element in the BDML schema (Table 1). The UnitKind element is based on the definition of the SBML schema (Hucka *et al*., 2003). We defined several additional units that are often used in experimental measurements and in computer simulations, such as a.u. (arbitrary unit) and micrometer. For example, fluorescence intensity in arbitrary units is used in many biological research projects and is included in the reported data (Bao *et al*., 2006; Keller *et al*., 2008; 2010; Sarov *et al*., 2012). We also predefined the units for temporal information under the tUnitKind element in the BDML schema; these units range from nanosecond to year (Table 2). More detailed information on the BDML schema is available online at http://ssbd.qbic.riken.jp/bdml/.

**Table 1.** Units for spatial and feature information predefined under UnitKind. The underlined words represent additional units, which are not defined in the SBML schema. The units a.u. and p.d.u. represent arbitrary unit and procedure defined unit, respectively

ampere	a.u.	becquerel	candela
Celsius	dimensionless	farad	gram
gray	henry	hertz	item
joule	katal	kelvin	kilogram
liter	liter	lumen	lux
meter	meter	micrometer	micrometer
mole	newton	ohm	pascal
p.d.u.	radian	siemens	sievert
steradian	tesla	volt	watt
weber

**Table 2.** Units for temporal information predefined under tUnitKind

nanosecond	microsecond	millisecond	second
minute	hour	day	month
year			

## 3 Results

### 3.1 Examples of BDML usage

We provide five detailed examples on how to describe object’s spatial information obtained from microscopic images or from computer simulation. These examples demonstrate that BDML can represent quantitative biological dynamics data from molecules to cells to organisms.

**Fig. 6.**
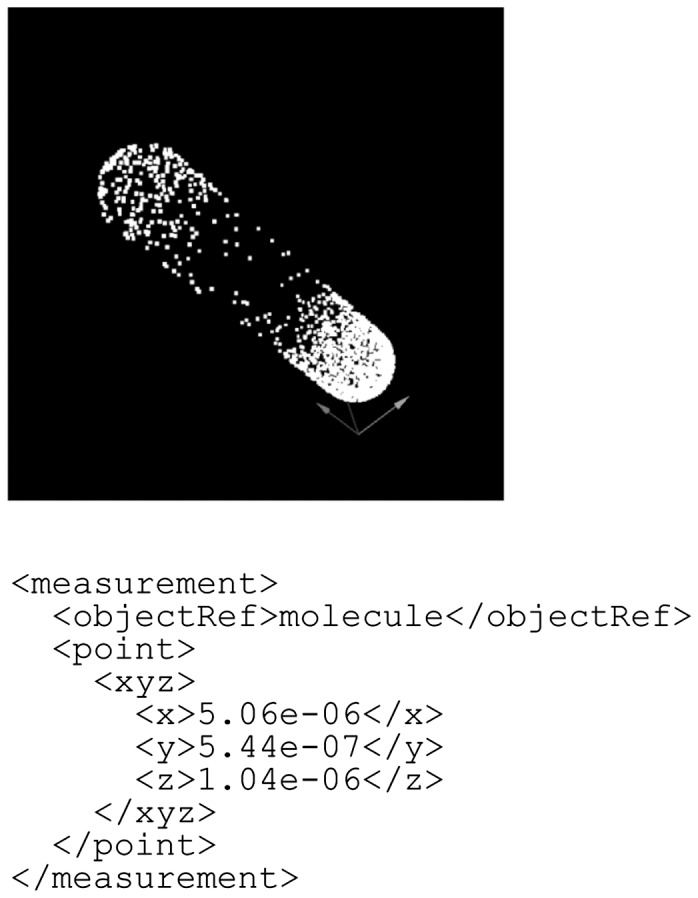
An example of single-molecule dynamics in *E.coli*. Each molecule is represented as a point. In this example, the units are in meters, and the scale factors for the *x*, *y* and *z* dimensions are set to 1.0

The first example is a computer simulation of single-molecule dynamics in *E.coli* (Fig. 6; Arjunan and Tomita, 2010), in which each molecule is represented as a point. Therefore, spatial information for a single molecule can be described by the point entity type in BDML. The coordinates of each molecule are described by the xyz element.

**Fig. 7.**
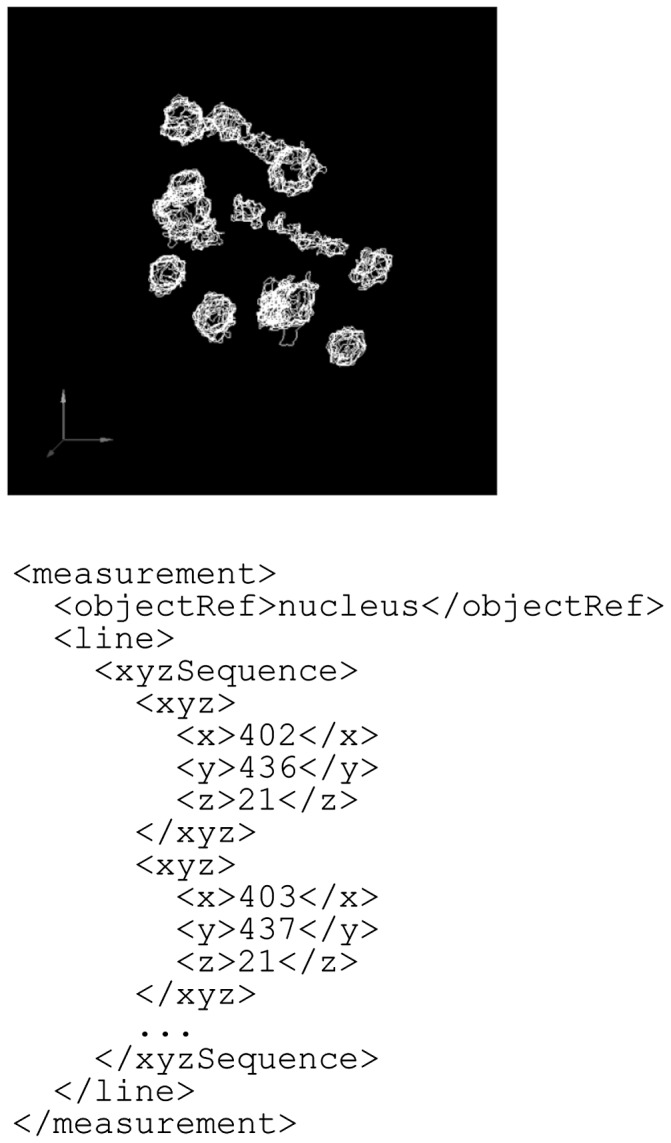
An example of cell division dynamics in a *C.elegans* embryo. Each nucleus is represented as a set of closed curves. In this example, the units are in micrometers, and the scale factors for the *x*, *y* and *z* dimensions are defined separately in the scaleUnit as 0.105, 0.105 and 0.5, respectively

The second example is an experimental measurement of cell division dynamics in a *C.elegans* embryo (Fig. 7). Kyoda *et al*. (2013) quantitatively extracted the dynamics of nuclear division by using differential interference contrast microscopy and image processing. Each nucleus is outlined by a set of closed polygonal chains, i.e. series of connected line segments with start point and end point joined together. The contour of a nucleus is therefore represented by the line entity type. In BDML, a closed polygonal chain is represented as a series of sequentially connected coordinates. The sequence of coordinates is described within the xyzSequence element.

**Fig. 8.**
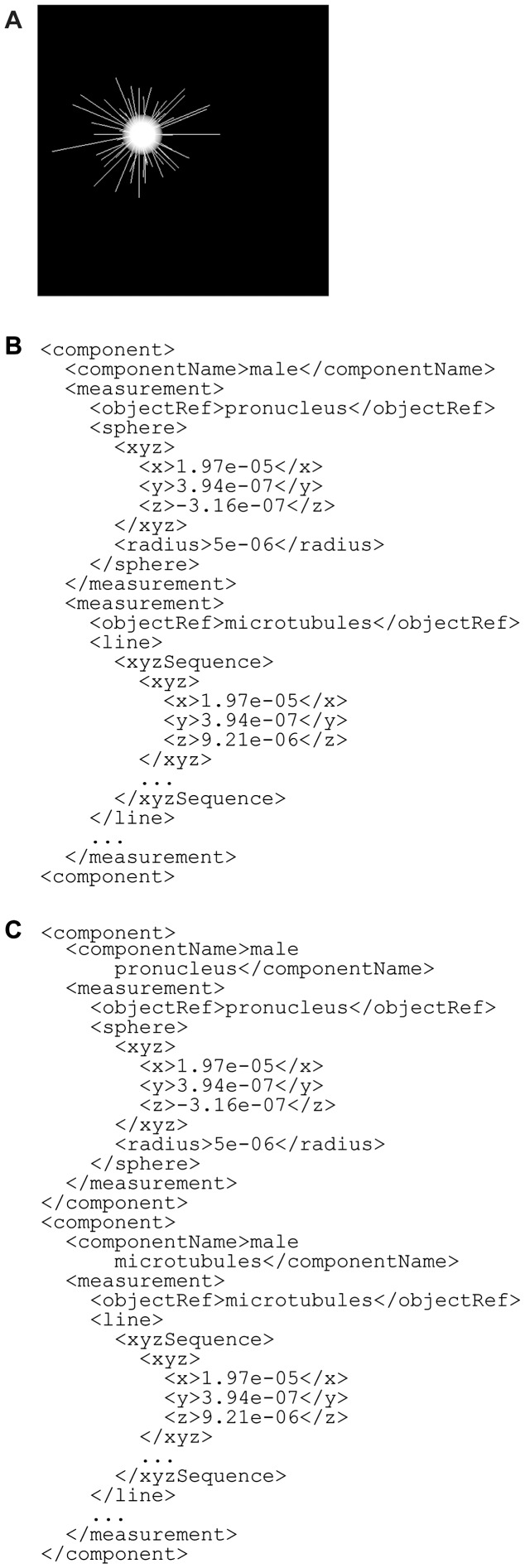
An example of dynamics of nuclear migration in a *C.elegans* embryo. The pronucleus and microtubules are represented as a sphere and a set of line segments, respectively (**A**). These structures can be represented as either one component (**B**) or as separate components (**C**). In this example, the units are in meters and the scale factors for the *x*, *y* and *z* dimensions are set to 1.0

The third example is a computer simulation of nuclear migration in a *C.elegans* embryo (Fig. 8; Kimura and Onami, 2005). The dynamics of the male pronucleus was predicted by calculating the dynamics of microtubules and their resultant forces on the pronucleus. The pronucleus is represented as a sphere and microtubules as a set of line segments. Spatial information for the pronucleus and microtubules can therefore be described using the sphere and line entity types, respectively. BDML has the flexibility to describe these objects as either one component or separate components (Fig. 8).

**Fig. 9.**
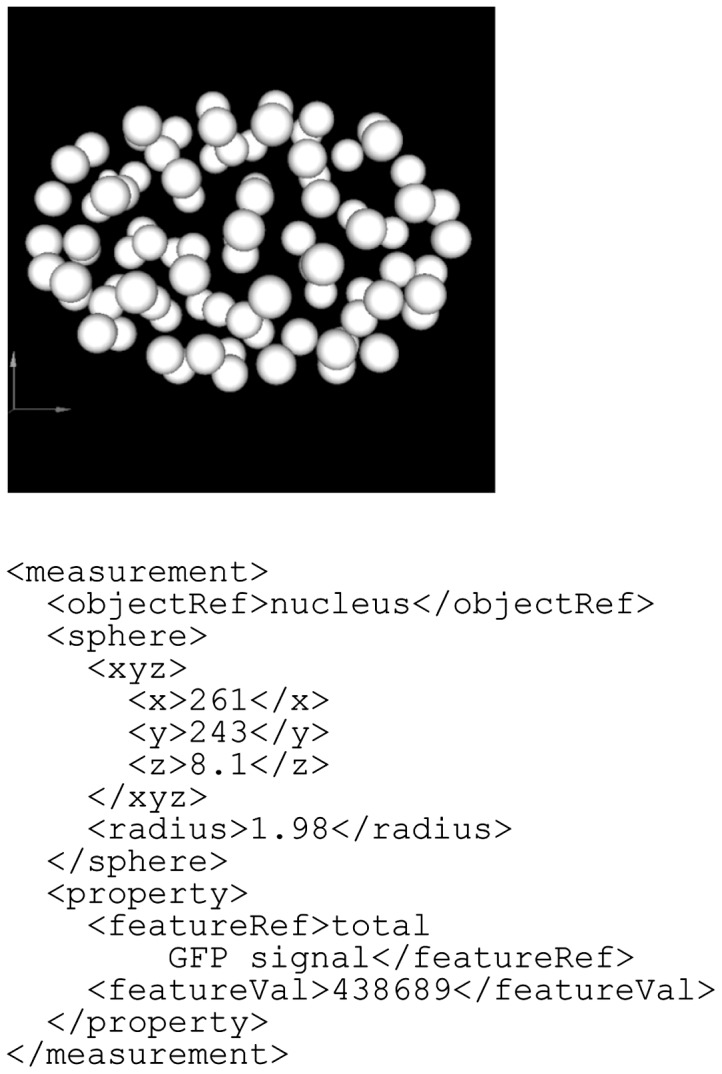
An example of gene expression dynamics at single-cell resolution in *C.elegans* embryo. Each nucleus is represented as a sphere, and total GFP signal is described in the property element. In this example, the units are in micrometers, and the scale factors for the *x*, *y* and *z* dimensions are defined separately in the scaleUnit as 0.9, 0.9 and 1.0, respectively

The fourth example is an experimental measurement of nuclear division and gene expression dynamics in a *C.elegans* embryo (Fig. 9; Bao *et al*., 2006). The dynamics of nuclear division was quantified by confocal microscopy and image processing. Each nucleus is represented as a sphere. Spatial information for the nucleus can therefore be described using the sphere entity type. Bao *et al*. (2006) also measured the expression dynamics of a gene encoding a GFP–histone fusion protein at single-cell resolution. BDML allows description of such a feature under the property element.

**Fig. 10.**
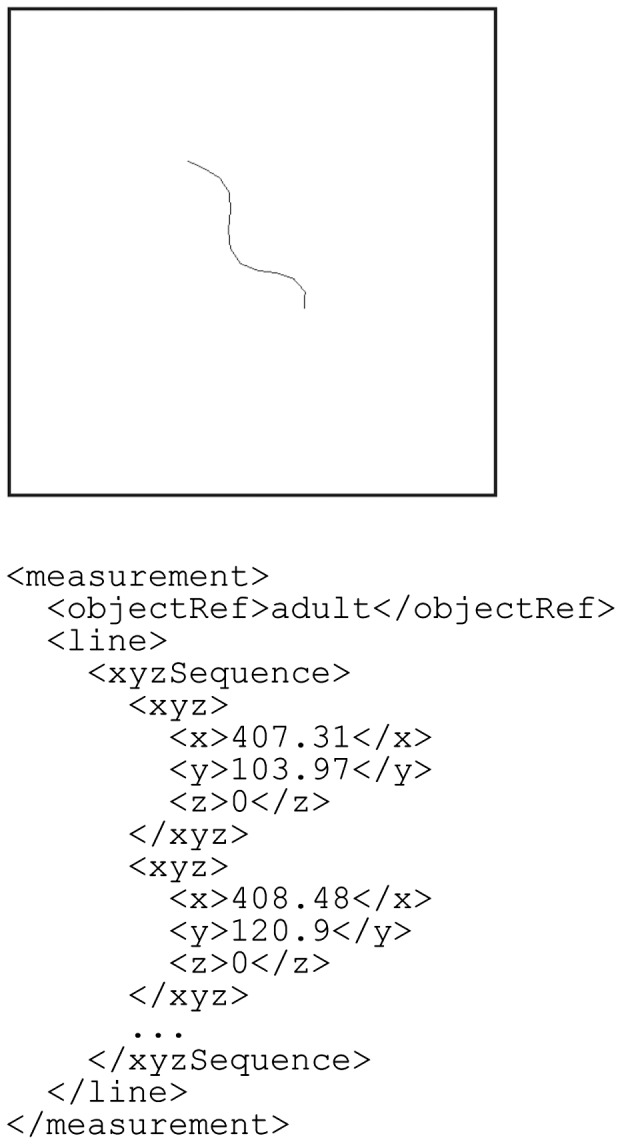
An example of behavioral dynamics of an adult *C.elegans*, represented as a set of line segments. In this example, the units are in micrometers and the scale factors for the *x*, *y* and *z* dimensions are defined separately in the scaleUnit as 4.1, 4.1 and 0, respectively

The final example is an experimental measurement of the behavioral dynamics of an adult *C.elegans* (Fig. 10). Cronin *et al*. (2005) quantitatively tracked the behavior of an individual worm, which is represented as a polygonal chain, i.e. series of connected line segments. Spatial information for the worm can therefore be described as the line entity type. In this case, the zScale element should be set to zero because the data were obtained from two-dimensional time-lapse microscopic images.

### 3.2 Software development

**Fig. 11.**
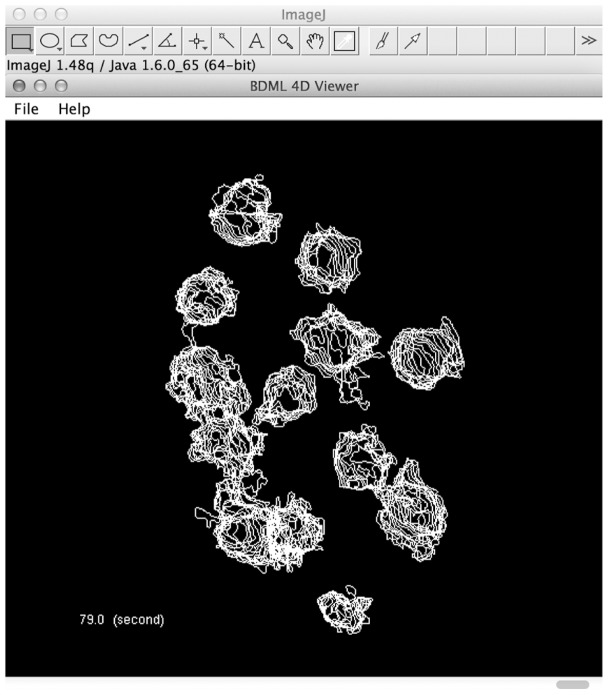
A snapshot of the BDML4DViewer software tool

The BDML format allows easy development of software tools, because a large number of open libraries and APIs for XML are freely available. To demonstrate this point, we developed a visualization software tool named BDML4DViewer. It reads BDML files and produces an onscreen interactive 4D visual representation of spatial information of the objects described in these files (Fig. 11). The user can view spatial information and scroll through different time frames interactively by using a mouse and keyboard. We developed this software tool as a plugin for ImageJ, a public-domain Java-based image processing application (Schneider *et al*., 2012), by using JAXB (Java Architecture for XML Binding) and JOGL (Java Binding for the OpenGL) APIs. This result demonstrates the ease of developing software tools using the BDML format. All source codes and the executable JAR file of BDML4DViewer are available online at http://ssbd.qbic.riken.jp/BDML4DViewer/.

## 4 Discussion

BDML is an open XML-based format for representing quantitative biological dynamics data. Although the five entity types used to describe dynamic spatial information (a set of points, a set of lines, a circle, a sphere and a set of faces) cover most of the publicly available data, other types of spatial entities (such as a cube or cylinder) can be potentially used. BDML can easily support these types of spatial entities by extending its format using XML with reference to geometric primitives commonly used in computer graphics such as X3D (Brutzman and Daly, 2007).

BDML provides a medium for representing a wide variety of quantitative data. Nearly 300 BDML datasets are currently available online at the Systems Science of Biological Dynamics (SSBD) database (http://ssbd.qbic.riken.jp). These datasets include dynamics of molecules, cells (nuclei) and gene expression and dynamics of whole organisms such as *E.coli*, *C.elegans*, *D.melanogaster* and zebrafish. Some of these datasets were derived from experimental measurements, whereas others were produced by computer simulation. BDML enables us to represent various types and scales of biological dynamics for different species.

Taking advantage of the BDML format, we developed and released BDML4DViewer for visualizing quantitative data from the open-source libraries and APIs. We are also developing software tools for extracting phenotypic characteristics from the data in the BDML format. Several of these tools are already available online at http://ssbd.qbic.riken.jp/phenochar/. The development of these software tools further demonstrates the flexibility of using BDML for visualization and analysis of different types of quantitative data. In addition, we believe that the BDML format would provide new opportunities for scientists in other fields such as statistics, physics and information science and facilitate bringing new ideas and approaches to biological analysis.

Besides its advantage as a human-readable structured data format, BDML also inherits some of the weaknesses of XML (Achard *et al.*, 2001; http://lw3.hdfgroup.org/projects/nara/XML_and_Binary.pdf). The numerical values of quantitative data are represented in BDML as decimal numbers in ASCII format. When numbers in double-precision floating-point format are written into a BDML file, storing the entire values may become impractical in terms of file size and file operations. Therefore, this representation method may result in truncation and loss of precision. BDML is hierarchical in structure, making it difficult to randomly access the data. The repetitive use of markup tags also makes it less efficient in terms of disk storage space than other data formats (Lawrence, 2004).

Opening or reading a BDML file using a software program can be a problem when the file size becomes too large. As BDML is a text-based hierarchical data format, large memory and computational resources are needed to parse the data file. To solve this problem, we included the series element, which can be used to divide a dataset into more than one BDML file. Using this element in the SSBD database enables practical use of the quantitative data for *D.melanogaster* and zebrafish embryos as series of BDML files.

A more general solution to the above problems would be to use another approach such as the eXtensible Data Model and Format (Clarke and Mark, 2007) or FieldML (Britten *et al.*, 2013). In these formats, the description of the data (light data) is separate from the numerical values (heavy data). The light data and heavy data are often stored in XML and HDF5 formats, respectively. This approach ensures numerical precision and reduces the required disk storage space and computational resources but lacks human readability. We are planning to expand BDML to also handle binary formatted data in the near future.

Although the ontology element is optional in the current version of BDML (0.2), it can provide unambiguous definitions of the terms in the BDML file. As most biologists are not familiar with ontological terms, tools for helping them to annotate the data in ontological terms at the time of file creation would be needed to make the option compulsory. Moreover, meta-information definition can be provided in the annotation element of the BDBase element by using the RDF. These definitions enable computer programs to understand the meaning of the terms and meta-information in the BDML file. The ontological references and annotations can be attached when the data are registered in some repository databases, such as SSBD.

A limitation of the current version of BDML (0.2) is the lack of hierarchical representation of meta-information about genetic perturbations (e.g. mutants, gene editing and RNAi treatments) and chemical perturbations (e.g. drug treatments). Such information would be useful for systematic comparison and analysis of biological dynamics by using more than one BDML file. Therefore, a future version of the BDML format will extend the BDML schema to support such information. As meta-information about genetic and chemical perturbations can be useful for other XML-based data formats such as MINiML (Barrett *et al*., 2007), mzML (Martens *et al*., 2011), OME (Allan *et al*., 2012), CellML (Hedley *et al.*, 2001), SBML (Hucka *et al*., 2003) and SBRML (Dada *et al*., 2010), we aim to collaborate with these projects to incorporate this information in a future release.

Integration and comparative analysis of various types of quantitative data are straightforward when they are represented in the BDML format. Such integration has the potential to lead to new insights into biological mechanisms; for example, an integrated study of the dynamics of cell morphology and protein activity has explored the relationship between biophysical phenomena and biochemical signaling (Tsukada *et al*., 2008). Comparison of experimental measurements and computer simulations has elucidated the mechanisms of various kinds of biological dynamics (Aliee *et al*., 2012; Grill *et al*., 2001; Kimura and Onami, 2005; Kozlowski *et al*., 2007; Krieg *et al*., 2008; Pecreaux *et al*., 2006; Rauzi *et al*., 2008; Stoma *et al*., 2011). BDML will facilitate such comparative analysis and comparison of the data from different laboratories. We believe that the BDML format will widen the range of scientific approaches to understanding biological systems.

## Supplementary Material

Supplementary Data
