# Supplementary material for: Biological Dynamics Markup Language (BDML): an open format for representing quantitative biological dynamics data
Source: Bioinformatics. 2014 Nov 19;31(7):1044–52. doi: 10.1093/bioinformatics/btu767 (PMC4382901; doi:10.1093/bioinformatics/btu767)
Supplement: Supplementary Data [file supp_31_7_1044__index.html]

Biological Dynamics Markup Language (BDML): an open format for representing quantitative biological dynamics data — Biological Dynamics Markup Language (BDML): an open format for representing quantitative biological dynamics data — Supplementary Data 

# Biological Dynamics Markup Language (BDML): an open format for representing quantitative biological dynamics data

## Supplementary Data

files

**Files in this Data Supplement:**

- Supplementary Data - doc file
