# Supplementary material for: Biological Dynamics Markup Language (BDML): an open format for representing quantitative biological dynamics data
Source: Bioinformatics. 2014 Nov 19;31(7):1044–52. doi: 10.1093/bioinformatics/btu767 (PMC4382901; doi:10.1093/bioinformatics/btu767)
Supplement: Supplementary Data [file supp_btu767_Kyoda_BDML_SupplementaryData.doc]

Supplementary Data

Biological Dynamics Markup Language (BDML): an open format for representing quantitative biological dynamics data

Koji Kyoda^1^, Yukako Tohsato^1^, Kenneth H. L. Ho^1^ and Shuichi Onami^1,2^,*

^1^Laboratory for Developmental Dynamics, RIKEN Quantitative Biology Center, Kobe 650-0047, Japan; ^2^National Bioscience Database Center, Japan Science and Technology Agency, Tokyo 102-0081, Japan

*To whom correspondence should be addressed

S1: Procedure for Data Processing Markup Language (PDPML)

PDPML elements

PDPML consists of four top-level elements: info, summary, contact and procedure. The info, summary and contact elements are similar to the corresponding elements in BDML. The specification and schema for PDPML are available at http://ssbd.qbic.riken.jp/pdpml/.

*Procedure*

An example procedure is as follows:

<procedure>

<description>

We simulated by E-cell3 (step order=1),

and extracted results (step order=2).

</description>

<step name="simulation process" order="1">

<annotation>

simulation of MinDE proteins in E. coli wild-type

(Arjunan and Tomita, 2010)

</annotation>

<program>

<name>E-Cell3 Spatiocyte</name>

<url>http://spatiocyte.readthedocs.org/en/latest/install.html.</url>

<description>

Installation guide for spatiocyte 1.0 and E-Cell System version 3 is

here: http://spatiocyte.readthedocs.org/en/latest/install.html.

</description>

</program>

</step>

<step name="selection process" order="2">

<annotation>selection of MinEE, MinDEE, MinDEED, and MinD</annotation>

<program>

<name>python programs for ecell4</name>

<url>https://github.com/ecell/ecell4/tree/master/export</url>

<description>

Selected molecules from the simulation results.

Targets: MinEE, MinDEE, MinDEED, and MinD, Log Interval 0.5(s)

</description>

</program>

</step>

</procedure>

The procedure element describes a procedure for obtaining quantitative data from the original sources such as *in vivo* microscopic images or files of the mathematical model for computer simulation. The description element summarizes information about the procedure. The procedure may consist of several independent steps. Each step can be described in the step element.

*Step*

The step element provides detailed information about each step of the procedure. The name of the step should be provided as an attribute of each step. In addition, the step order can be provided. The annotation element summarizes information about the step. The program used in the step should be described in the program element.

*Program*

The program element describes information about the program used in each step of the procedure. The name of the program should be provided in the name element. The version number and the name of the program and its download link can be provided in the elements version and url, respectively. Detailed information about configuration and parameters used in the program can be provided in the description element.
